# Report of the sixth meeting of the European Consortium ‘Care for CMMRD’ (C_4_CMMRD), Paris, France, November 16th 2022

**DOI:** 10.1007/s10689-024-00403-1

**Published:** 2024-07-20

**Authors:** Léa Guerrini-Rousseau, Richard Gallon, Marta Pineda, Laurence Brugières, Stéphanie Baert-Desurmont, Carole Corsini, Volodia Dangouloff-Ros, Mark A. J. Gorris, Christine Haberler, Pauline Hoarau, Marjolijn C. Jongmans, Matthias Kloor, Jan Loeffen, Charlotte Rigaud, Julie Robbe, Roseline Vibert, Dilys Weijers, Katharina Wimmer, Chrystelle Colas

**Affiliations:** 1 Department of Pediatric and Adolescent Oncology, Gustave Roussy Cancer Campus, Université Paris-Saclay, Villejuif, France; 2 Molecular Predictors and New Targets in Oncology, INSERM U981, Gustave Roussy, Université Paris-Saclay, 114 Rue Edouard Vaillant, 94805 Villejuif, France; 3 Translational and Clinical Research Institute, Faculty of Medical Sciences, Newcastle University, Newcastle upon Tyne, UK; 4 Hereditary Cancer Program, Catalan Institute of Oncology-IDIBELL, Barcelona, Spain; 5 Inserm U1245, Univ Rouen Normandie, Rouen, France; 6 Department of Genetics, CHU Rouen, Rouen, France; 7 Medical Genetics Department, Centre Hospitalier Regional Universitaire de Montpellier, Montpellier, France; 8 Pediatric Radiology Department, Hôpital Necker Enfants Malades, AP-HP, Paris, France; 9 UMR 1163, Institut Imagine and INSERM U1299, Université Paris Cité, Paris, France; 10 Department of Medical BioSciences, Radboud University Medical Center, Nijmegen, The Netherlands; 11 Division of Immunotherapy, Oncode Institute, Radboud University Medical Center, Nijmegen, The Netherlands; 12 Division of Neuropathology and Neurochemistry, Department of Neurology, Medical University of Vienna, Vienna, Austria; 13 Princess Máxima Center for Pediatric Oncology, Utrecht, The Netherlands; 14 Department of Genetics, University Medical Center Utrecht, Utrecht, The Netherlands; 15 Department of Applied Tumour Biology, Institute of Pathology, Heidelberg University Hospital, and Clinical Cooperation Unit Applied Tumor Biology, German Cancer Research Center, Heidelberg, Germany; 16 Division of Hemato-Oncology, Princess Máxima Center for Pediatric Oncology, Utrecht, The Netherlands; 17 Department of Genetics, Institut Curie, PSL University, Paris, France; 18 Department of Genetics, Hôpital Pitié-Salpêtrière, AP-HP, Sorbonne Université, Paris, France; 19 Institute of Human Genetics, Medical University of Innsbruck, Innsbruck, Austria

**Keywords:** CMMRD, European C_4_CMMRD consortium, C_4_CMMRD, Meeting report

## Abstract

Biallelic germline pathogenic variants in one of the four mismatch repair genes (*MSH2*, *MSH6*, *MLH1* and *PMS2*) cause a very rare, highly penetrant, childhood-onset cancer syndrome, called constitutional mismatch repair deficiency (CMMRD). The European consortium “Care for CMMRD” (C4CMMRD) was founded in Paris in 2013 to facilitate international collaboration and improve our knowledge of this rare cancer predisposition syndrome. Following initial publications on diagnostic criteria and surveillance guidelines for CMMRD, several partners collaborating within the C4CMMRD consortium have worked on and published numerous CMMRD-related clinical and biological projects. Since its formation, the C4CMMRD consortium held meetings every 1–2 years (except in 2020 and 2021 due to the Covid 19 pandemic). The sixth C4CMMRD meeting was held in Paris in November 2022, and brought together 42 participants from nine countries involved in various fields of CMMRD healthcare. The aim was to update members on the latest results and developments from ongoing research, and to discuss and initiate new study proposals. As previously done for the fifth meeting of the C4CMMRD group, this report summarizes data presented at this meeting.

## Introduction

Germline pathogenic variants (PV) in one of the four mismatch repair (MMR) genes (*MSH2*, *MSH6*, *MLH1* and *PMS2*) are associated with increased cancer risk. Heterozygous MMR PV cause Lynch syndrome (LS), one of the commonest, adult-onset cancer syndromes, whilst biallelic MMR PV, first described in 1999, cause a rare, childhood-onset cancer syndrome called constitutional mismatch repair deficiency (CMMRD) [1, 2]. CMMRD patients have a very high risk for a wide variety of malignant neoplasms occurring as early as the first year of life, most prevalently cerebral, gastrointestinal, and hematologic malignancies [3, 4].

The European Consortium “Care for CMMRD” (C4CMMRD) was founded in Paris in 2013 to facilitate international collaboration and improve our knowledge of this rare cancer predisposition syndrome. An early achievement of the consortium was to publish diagnostic criteria [3] and surveillance guidelines [5] for CMMRD. Since then, several collaborating partners within the C4CMMRD consortium have worked on and published numerous clinical and biological projects related to CMMRD [3, 5–10] (Rigaud et al., under review), and the C4CMMRD Database has been created as a registry for CMMRD patients [11].

Since its formation, the C4CMMRD consortium held meetings every 1–2 years until 2019. Due to the Covid 19-pandemic, international meetings had to be suspended through 2020 and 2021. The 6th C4CMMRD meeting was held in Paris in November 2022 with the aim of updating members on the latest results and developments from ongoing research, and initiating new study proposals. A half-day educational session on CMMRD was also given at the SIOPe Host Genome Working Group (HGWG) meeting, the day before the C4CMMRD meeting. Forty-two participants from nine countries and various medical fields (including basic and translational researchers, pediatric oncologists, clinical geneticists, genetic counselors, gastroenterologists, molecular geneticists and one psychologist) attended the 6th C4CMMRD meeting. This report summarizes data presented at this meeting.

## The C4CMMRD Database

The C4CMMRD Database was set-up in 2014 to collect clinical, familial and genetic data from European patients with CMMRD. Initially, data were collected using a paper Case Report Form (CRF) format. Pauline Hoarau, the project manager in charge of the C4CMMRD Database, presented changes made recently to better facilitate access and contribution to the database. The C4CMMRD Database is now fully electronic with an electronic CRF (e-CRF) and pseudonymized data, using REDCap [12, 13] electronic data capture tools, whose servers are hosted in Ile de France by Claranet, an approved healthcare data host.

Different types of access to the database are possible. Data can be registered from any place in the world through a secure web connection. Direct access of each participating center to its patients’ data is possible after authentication by an ID and a password. Extracts of summary statistics and more exact data queries can also be performed depending on the user rights. For specific projects utilizing the database, access and modalities will be given to the project lead after review and approval of a project proposal by the Steering Committee.

Retrospective data, collected so far in paper form, have been transferred into the electronic database and participants and/or their legal guardian(s) have been informed of this change to data storage and management. For prospective data collection, a consent has to be obtained from the patient and/or their legal guardian(s) before registration. The new database is approved by INSERM French Ethical committee (20_681) and complies with French Data Protection Authority (CNIL) requirements (INDS Protocol No.: 1008261119). In each country, a coordinator will have to ensure the conformity of this project with their national regulations.

Data from 101 patients from 16 countries have been collected so far, allowing work on different aspects of CMMRD including cancer risk in relatives [7], CMMRD-associated brain tumors [8], CMMRD-associated NHL (Rigaud et al., under review), and screening for tumors in CMMRD patients [10]. Several new projects are ongoing or will be set up within a few months.

Any site/user wishing to participate and to register a new patient can receive access and an informed consent form by contacting the project coordinator at c4cmmrd@gustaveroussy.fr.

## Improved ancillary tests for CMMRD diagnostics and screening

Identifying paediatric cancer patients suspected to have CMMRD currently uses clinical criteria [3] that were designed for high sensitivity and, consequently, have low specificity. Subsequent genetic diagnosis can be complicated by interpretation of MMR variants of uncertain significance (VUS) and the detection and interpretation of *PMS2* variants can be complicated due to its pseudogenes. Ancillary tests of MMR function in constitutional tissues complement genetic testing for CMMRD [3]. Notable examples that have high sensitivity and specificity for CMMRD detection include ex vivo microsatellite instability (MSI) analysis and methylation tolerance assays of patient-derived lymphoblastoid cell lines [14], and MSI analysis of patient peripheral blood leukocytes using targeted amplicon sequencing [6, 15], or whole genome sequencing [16, 17].

Marta Pineda (Catalan Institute of Oncology-IDIBELL, Barcelona, Spain) presented a national collaborative project, co-led by Luis Ignacio González-Granado (Hospital Universitario 12 de Octubre, Madrid, Spain) and comprising an interdisciplinary group of experts in CMMRD from 22 Spanish institutions, that aims to improve identification of CMMRD patients in the Spanish population. Marta Pineda also provided new results obtained from the previously published high sensitivity MSI (hs-MSI) assay [15] following its use in a blinded cohort of 66 blood samples (from 33 confirmed CMMRD individuals, 22 non-CMMRD controls and 11 CMMRD-suspected cases) and 24 CMMRD tumours, in a collaboration with the International Replication Repair Deficiency Consortium (IRRDC). hs-MSI analysis of blood samples displayed high diagnostic accuracy for CMMRD (sensitivity 98.5% and specificity 100%), also detecting MSI in all CMMRD-associated tumours [18]. Finally, Marta Pineda presented the results obtained with a newly developed approach, hs-MSI32, which assesses a smaller panel of 32 MSI markers (compared to the original hs-MSI panel of 186 MSI markers) that were found to be recurrently unstable in CMMRD patients’ blood. The validation of this novel approach using a blinded cohort of 80 blood samples from the European C4CMMRD consortium, reached 96% sensitivity and 98% specificity. Of note, hs-MSI and hs-MSI32 tools are being implemented in the Molecular Diagnostic Laboratory at the Catalan Institute of Oncology (Barcelona, Spain).

Richard Gallon (Newcastle University, UK) presented a recently published constitutional microsatellite instability (cMSI) assay that can detect CMMRD with high accuracy [9]. The cMSI assay was developed by enhancing an existing amplicon-sequencing method [6] with novel markers selected specifically for cMSI analysis of blood samples. Candidate MSI markers were identified by genome sequencing of three CMMRD (one *MSH6*- and two *PMS2*-associated), one LS (*MLH1*-associated), and two control blood samples. Using amplicon sequencing of a pilot cohort of eight CMMRD and 38 control blood samples, these were refined to a panel of 32 highly sensitive markers for cMSI analysis. The enhanced cMSI assay was used to analyze a blinded cohort, comprising 56 CMMRD, 43 control, and 8 CMMRD-negative (patients suspected of CMMRD where no germline MMR variant was found) blood samples, as well as blood samples from 40 known LS carriers. Eighty, independent reference control blood samples provided reference data for cMSI score calculation. The CMMRD samples had much higher cMSI scores [range 20.9–300.0] than controls [range 0.0–3.6], LS [range 0.0–11.3], and CMMRD-negative [range 0.2–5.3] samples, allowing unambiguous classification. cMSI scores were reproducible on repeat testing (*R* = 0.994, *p* < 10^−15^). Among CMMRD samples, cMSI score was associated with the germline-affected gene (*p* = 1.2 × 10^−3^), with MSH6-deficient patients having lower cMSI scores than each of MLH1- (*p* = 0.05), MSH2- (*p* = 2.4 × 10^−4^), and PMS2-deficient (*p* = 6.0 × 10^−3^) patients. Lower cMSI scores were observed in patients carrying at least one missense variant (*p* = 7.4 × 10^−3^), whilst patients with biallelic truncating and/or copy number variants had higher cMSI scores (*p* = 0.02). cMSI score, however, did not correlate with age of first cancer (*ρ* = −0.154, *p* = 0.29), suggesting it would not be a clinically-useful predictor of cancer risk in CMMRD patients. The method is low cost, scalable, and fully automatable, making the cMSI assay suitable for high throughput molecular screening.

## Resolving diagnosis in CMMRD-LIKE or atypical CMMRD patients

### Differential diagnoses associated with replication repair deficiency in patients with a CMMRD-like phenotype

One of the main tasks of MMR is the correction of replication errors that escape proofreading by the polymerases ε and δ (Pol ε, Pol δ) [19–22]. MMR deficiency (MMRD) and polymerase proofreading defects (PPD) are observed in a variety of tumor types. MMRD and PPD are both associated with high tumor mutational burden (TMB) and specific COSMIC mutational signatures. Whilst MMRD is caused by a broad spectrum of variants that disrupt function of both alleles of an MMR gene, PPD is caused by heterozygous missense variants inactivating the exonuclease activity of Pol ε or Pol δ encoded by the genes *POLE* and *POLD1*, respectively. MMRD and PPD, separately or combined, are also observed in tumors of patients with CMMRD, LS, or polymerase proofreading-associated polyposis (PPAP). PPAP is caused by heterozygous germline *POLE* or *POLD1* PV and is associated with gastrointestinal polyposis, as well as adult-onset colorectal and endometrial cancer, and other tumor types [23]. Of note, concurrent MMRD and PPD is seen in the majority of CMMRD-associated malignant brain tumors and approximately one third of CMMRD-associated gastrointestinal cancers, due to the biallelic germline MMR PV along with somatic *POLE*/*POLD1* PV [24–27]. The specific COSMIC signatures of these combined replication error repair defects were characterized by Haradhvala et al. [28].

Katharina Wimmer (Medical University Innsbruck, Austria) presented possible differential diagnoses of CMMRD that result from constitutional deficiencies in replication repair. These cases benefitted from the availability of assays of MMR function in normal tissues to complement genetic testing [6, 9, 14, 15, 17] that can reliably confirm or refute a diagnosis of CMMRD.

One possible differential diagnosis is PPAP that is caused by specific, highly penetrant germline variants leading to an exceptionally early manifestation in childhood rather than the typical adult onset of cancer. In five published patients fulfilling C4CMMRD criteria to indicate CMMRD testing [3], including malignant and non-malignant features, the CMMRD-like phenotype was explained by a heterozygous *POLE* germline PV [29–31]. Of note, none of these five *POLE* variants have been previously identified in the germline of patients with PPAP, but they are all reported as somatic driver mutations in hyper-mutated (TMB > 10 mut/Mb) tumors [21, 26, 32]. This suggests that the *POLE* variants found in CMMRD-like patients lead to a more severe phenotype than PPAP-associated *POLE* variants, possibly as a result of a stronger “mutator” effect.

Another possible differential diagnosis is a digenic syndrome caused by concurrent heterozygous germline PV in both polymerase exonuclease domains and MMR genes. In two recently published siblings with polyposis and a first colorectal cancer diagnosis as teenagers, a heterozygous *PMS2* PV and a heterozygous *POLD1* exonuclease domain variant were found. The presence of the COSMIC mutational signature linked to combined MMRD and Pol δ proofreading deficiency in their tumors confirmed that these germline variants together explain the CMMRD-like phenotype of these siblings [33]. Other cases include the finding of heterozygous *PMS2* and heterozygous *POLE* PV in the germline of a patient with a similar phenotype of teenage-onset colorectal cancer [34] and in the germline of a patient derived from a PPAP family with a sonic-hedgehog medulloblastoma at the age of 4.5 years [35]. Together, these cases constitute a new digenic cancer predisposition syndrome that should also be considered as a differential diagnosis in patients with a CMMRD-like or a severe LS phenotype.

A study presented by Roseline Vibert (Paris, France) investigated more broadly whether additional germline variants in cancer susceptibility genes may explain “extreme LS-phenotypes”, i.e. cancer before the recommended screening age of LS patients that may lead to suspicion of CMMRD. This study compared the prevalence of additional cancer predisposition gene variants in LS patients with a cancer diagnosis before age 30 (early-onset group, *n* = 43 patients) and after age 40 (usual-onset group, *n* = 92 patients) [36]. An excess of PV and VUS were found in early-onset cases when only gastrointestinal cancer susceptibility genes were considered (OR 2.25; 95% CI: 1.01–5.06, *p* value = 0.04). Four early-onset cases stood out: two with *POLE*/*POLD1* exonuclease domain variants, one with a *BMPR1A* duplication and one with an *EPCAM* deletion. In particular among these four cases, one teenage female aged 17, carrying a *PMS2* pathogenic variant and a *POLE* VUS located in the key exonuclease domain, presented with a duodenal adenocarcinoma with a high TMB (111 mutations/Mb) and with the SBS14 MMR/POLE signature, in favor of the involvement of both variants in carcinogenesis.

### CMMRD with Lynch syndrome-like presentation

An attenuated form of CMMRD, typically presenting with LS-associated cancers and often associated with first cancer diagnoses in adulthood rather than childhood or adolescence, has been associated with a *PMS2* founder variant in the Nunavik population [37]. This variant (*PMS2* c.2002A>G) causes mis-splicing and truncation of *PMS2* (p.Ile668*) but has residual expression of full length *PMS2* transcript and protein containing a missense variant (p.Ile668Val). Reduced rather than ablated PMS2 function in *PMS2* c.2002A>G homozygotes likely explains their attenuated CMMRD phenotype. This raises the possibility that attenuated forms of CMMRD could be linked to other MMR PV.

Katharina Wimmer (Institute of Human Genetics, Innsbruck, Austria) added to this notion by reporting a patient with an endometrial cancer in her late 30 s who was found to be homozygous for an *MLH1* variant c.306G>A, which is reported by InSiGHT as a VUS. *MLH1* c.306G>A is a synonymous variant (p.(Glu102=)) but was shown through transcript analysis to disrupt splicing leading to in-frame loss of exon 3 in transcripts and 33 amino acids of the MLH1 protein. However, like *PMS2* c.2002A>G, the splice effect of *MLH1* c.306G>A is not complete as residual full length transcript was detectable in the patient’s tissues. CMMRD was confirmed using constitutional MSI analysis [9]. This patient and another novel case of late onset CMMRD, along with an analysis of associated genotypes and phenotypes using these and additional cases identified by literature review, have recently been published (Gallon et al., in press).

These cases suggest that attenuated forms of CMMRD with a Lynch-like phenotype may be caused by homozygosity or compound heterozygosity of hypomorphic MMR gene germline variants. To better understand this phenotype of late-onset CMMRD, which has implications for MMR variant classification and patient management, Richard Gallon suggested collecting and evaluating more CMMRD patients with this phenotype in a collaborative C4CMMRD project.

### A probable CMMRD case with an uncertain diagnosis

The differential diagnoses of CMMRD-like patients and atypical cases of CMMRD supports the concept of a phenotypic continuum between CMMRD, LS, and other replication repair deficiency-associated cancer predisposition syndromes such as PPAP. These overlaps can complicate diagnosis, especially where genetic testing does not provide a definite result.

Stéphanie Baert-Desurmont (CHU Rouen, France) reported a 51 year old patient with 2 café au lait macules, who presented with several malignant tumors from the LS spectrum (caecal, sigmoid and rectal carcinomas) from the age of 17 years [38]. The patient also had pancreatic and renal tumors, and two ureteric tumors without MSI but with a loss of MSH6 expression by immunohistochemistry (IHC) on ureteral tumour cells and weak expression on tumour-adjacent cells. Genetic testing revealed the patient is compound heterozygous for two *MSH6* variants, a class 5 PV c.3261dup and a VUS c.2561_2563del. No PV were found in other genes associated with colorectal cancer and/or polyposis, including *POLE*/*POLD1* genes. Ancillary functional tests combining MSI from lymphoblastoid cell lines and tolerance to methylating agents [14] were inconclusive (evMSI normal and MT in favor of CMMRD). Despite CMMRD not being confirmed, it remains the most likely diagnosis in this patient and additional ancillary tests would be useful to resolve this uncertainty.

## Immunological and molecular features of CMMRD tumors

### Involvement of the immune system in CMMRD tumorigenesis

MMRD triggers the accumulation of high numbers of somatic mutations. These mutations are mainly insertions/deletions (indels) [16] at repetitive microsatellite sequences. Indels in coding microsatellites can lead to translation frameshifts and the generation of immunogenic neopeptides [39], frameshift-derived peptides (FSP), which can be recognized by the host’s immune system. As these FSPs do not undergo thymic selection for tolerance, they are thought to be highly immunogenic and ideal candidates for immune-directed therapies [40, 41]. Therefore, immune surveillance plays a major role in inherited conditions that predispose to the development of MMRD tumors. FSPs are produced by the MMRD cells of LS-patients that have acquired a second hit in the germline-affected MMR gene. In contrast, CMMRD is characterized by MMRD in all somatic cells. Therefore, whilst the role of the immune system is of great interest in both syndromes, it may have distinct effects in CMMRD tumorigenesis.

Matthias Kloor (Heidelberg University Hospital, Germany) presented a collaborative study with Steven Lipkin (Weill Cornell University, New York) and the National Cancer Institute. They used mouse models with MMRD intestinal epithelium (VcMsh2^−/−^ mice) to analyze the effects of vaccination with recurrent MMRD-induced FSPs on tumorigenesis, showing that FSP vaccination prolonged survival and reduced tumor burden [42]. In addition, their recent observations from human colorectal epithelium suggest that local immune surveillance mechanisms can control tumor outgrowth in LS [43], and therefore similar mechanisms may exist within CMMRD.

Mark A. J. Gorris (Radboud University Medical Center, Nijmegen, The Netherlands) presented a clinical trial (NCT01885702) in which 23 LS patients were vaccinated with dendritic cells loaded with FSPs derived from two genes: transforming growth factor beta receptor 2 (TGFβR2) and caspase 5 (CASP5). After vaccination, FSP-specific T-cells were detected for TGFβR2 and CASP5 and were able to eradicate FSP-loaded tumor cell lines.

Following this result, their team is studying which FSPs are shared between different tumor types and individual patients within a cohort of CMMRD patients. They sequenced DNA of 42 tumors (14 brain malignancies, 13 GI-tract tumors, 2 adenoma’s and 13 hematological malignancies) derived from 17 CMMRD patients and identified over 6,000 frameshift mutations (of which more than 1,000 occurred in at least 2 different patients). Currently, they are investigating HLA binding peptides that are determined by in silico prediction programs (NetChop, NetpanMHC, and MHCFlurry). Predicted peptides will be tested for their binding capacity empirically. In addition, blood of CMMRD patients will be analyzed for pre-existing immune responses against binding FSPs.

### Deciphering the molecular characteristics of CMMRD-associated tumors

The contribution of MMRD to cancer development has been acknowledged now for more than two decades. Nevertheless, identifying the exact mechanisms of oncogenesis in these tumors is a fundamental issue, with the aim of understanding elements that can influence the tumor’s mutational landscape and possibly discovering specific mutational events that could become new targets for treatment and prevention. Somatic mutations identified after performing tumor whole genome or exome sequencing (WGS/WES) allow determination of the tumor mutational load and signatures.

Dilys Weijers (Princess Máxima Center, Utrecht, The Netherland’s) presented the result of molecular features analysis of 41 tumors from a cohort of 16 Dutch CMMRD-patients: brain (*n* = 14), gastrointestinal tract (*n* = 12) and hematological (*n* = 13) malignancies, as well as two adenomas. Contributions of signatures associated with MMRD (SBS6, SBS15 and SBS26) and concurrent defects in the exonuclease domain of *POLE* and *POLD1* (SBS14 and SBS20) were found in the CMMRD tumors, depending on the affected gene and tumor type. Additionally, they found contributions of temozolomide treatment-associated signature SBS11 in two hematological malignancies. This finding raises the question whether temozolomide treatment of brain tumors in children with CMMRD is not only ineffective but could even play a role in the development of new hematological malignancies.

Léa Guerrini-Rousseau (Gustave Roussy Cancer Campus, Villejuif, France) reported clinical, histopathological and genomic data from 12 CMMRD-patients with a high-grade glioma (HGG) (10 glioblastomas and 2 anaplastic astrocytomas) (Guerrini-Rousseau et al., under review). After somatic WES analysis, they reported that in most of the 12 patients but not all, the glioma harbored an ultra-hypermutated phenotype (>100 single nucleotide variants per megabase (SNV/Mb)). No correlation was observed between the number of mutations and sex, age, survival or mutated MMR gene. Driver mutations in *POLE* and *POLD1* exonuclease domains were described for 8 and 1 patients respectively. To characterize the impact of the *POLE*/*POLD1* driver mutation on the mutation load, the number of mutations and signatures occurring before and after its occurrence were analyzed separately, assuming that mutations with higher variant allele frequency (VAF) than the VAF of the driver *POLE*/*POLD1* mutation would have occurred before it. These analyses suggest that the ultra-hypermutation phenomenon may start before the occurrence of the polymerase driver mutation. First, four patients had more than 100 mutations/Mb with higher VAF than the identified *POLE* driver mutation. Second, *POLE*/*D1* driver mutations did not contribute to a significant increase in mutational load between the mutations with a lower VAF than the *POLE*/*POLD1* driver mutation compared with mutations with higher VAFs, except in one patient. Third, the mutation signatures were dominated by MMRD-related mechanisms and were similar in the different mutation bursts of the same patient, without enrichment of PPD-related mutational signatures in later bursts. This report also highlighted that CMMRD-associated gliomas have a distinctive oncogenesis that does not involve the usual pathways and mutations seen in sporadic pediatric or adult glioblastomas. Of note, *SETD2*, *TP53*, *NF1*, *EPHB2*, *ATRX* and *DICER1* genes were frequently mutated in CMMRD gliomas and could be secondary driver mutations.

## Features of specific tumor types associated with CMMRD

### Brain tumors

#### Neuroimaging features of CMMRD and brain MRIs of children with high-grade gliomas according to MMR gene mutation status

Alongside the high risk of HGG, several other brain abnormalities detected on brain imaging have been reported in CMMRD patients. Volodia Dangouloff-Ros (Necker hospital, Paris, France) presented an overview of the radiological features usually found on brain MRIs of CMMRD patients. The brain MRI of 30 CMMRD-patients (with or without HGG) were compared to MRI of children with non-CMMRD related HGG (7 LS, 4 NF1 and 50 sporadic HGG). HGG in CMMRD and LS patients were predominantly hemispheric (versus midline) compared to NF1 and sporadic HGG. CMMRD-associated tumors often had ill-defined boundaries. All CMMRD patients had at least one developmental venous anomaly (DVA) (30/30, 100%), which were found in only 14% of LS patients (1/7), and 6% of sporadic HGG patients (3/50) (*p* < 0.0001). Multiple DVAs (at least 2) were found in 83% of CMMRD patients, one NF1 patient (3%) and not in other groups (*p* < 0.0001). Cavernomas (before radiotherapy) were only found in CMMRD patients (21% of the cases, *p* = 0.008). NF1-like focal areas of high FLAIR signal intensity (FASI) were more frequent in CMMRD patients than in LS or sporadic patients (50%, vs. 20% and 0%, respectively, *p* < 0.0001). Data on specific brain MRI features of CMMRD in children with HGGs will be published soon (Dangouloff-Ros et al. in press).

#### Defects of mismatch repair proteins in pediatric high grade gliomas: Austrian data

Christine Haberler (Medical University of Vienna, Austria) reported the results of a combined approach using IHC and molecular analyses by NGS in tumour tissue from an Austrian cohort of 50 pediatric HGG-patients to improve the detection of CMMRD and LS patients. By IHC, loss of PMS2 or combined MSH2/MSH6 loss was found in 5 patients with genetically confirmed CMMRD and diffuse pediatric-type high-grade glioma, H3 wildtype and IDH-wildtype. This method identified 3 additional patients with a somatic MMR mutation in a diffuse pediatric-type, H3-wt, and IDH-wt HGG. The Austrian team proposes that this type of combined approach could be proposed in all such cases.

### Hematological malignancies

#### Analysis of a large series of CMMRD-associated lymphomas

Charlotte Rigaud (Gustave Roussy Cancer Campus, Villejuif, France) presented data on characteristics and outcomes of a large series of CMMRD-associated lymphomas diagnosed before 2020. Data from this series of patients could be collected thanks to a collaboration between C4CMMRD, the IRRDC and the European Intergroup for Childhood Non-Hodgkin lymphoma (EICNHL).

Overall, 74 CMMRD patients diagnosed with a total of 100 NHL were identified with a median age at diagnosis of the first NHL of 9.4 years. A previous malignancy was reported in 26 patients (35%) and 20 patients (27%) had multiple lymphomas, including 14 with 2 NHL and 6 with 3 NHL. The first NHL was a T-cell lymphoblastic lymphoma (T-LBL) in 53 cases (72%), a precursor B-lymphoblastic lymphoma in 4 cases (5%) and a mature B-NHL in 17 cases (23%), including 10 diffuse large B-cell lymphomas, 6 Burkitt lymphomas, and one other high-grade B-cell lymphoma. All patients were treated with current chemotherapy regimens adapted to subtype. Standard treatments for sporadic NHL appeared to be effective in most CMMRD-associated NHL as, with a median follow-up of 8.7 years, the 5-year cumulative risk of progression/relapse after the first NHL was 20.8%. This is considered acceptable in this group of patients treated over a long period of time. However, the prognosis was impaired by the high risk of second malignancies, including multiple lymphomas, with a cumulative risk of second malignancy reaching 52.9% at 5 years. The role of chemotherapy in the pathogenesis of these secondary malignancies has not been assessed yet, and specific trials should be organized in this population to test new therapeutic approaches to limit genotoxic treatment.

#### High prevalence of constitutional mismatch repair deficiency in a pediatric T-cell lymphoblastic lymphoma cohort

Jan Loeffen (PMC, Utrecht, The Nederland’s) presented results from the Dutch cohort of 88 unselected T-cell Lymphoblastic Lymphoma (T-LBL) patients, including 8 CMMRD patients (biallelic germline mutations in *PMS2* or *MSH6*). They compared the findings on clinical presentation of CMMRD-associated T-LBL patients to sporadic T-LBL patients. Overall, disease presentation was comparable. However, the disease might be more localized in CMMRD-associated T-LBL: 9% of the sporadic T-LBL patients presented with 5% or more blasts, and an additional 19% with 1–4% blasts, in the bone marrow, whereas none of the CMMRD patients had any blasts in the bone marrow. Additionally, none of the CMMRD patients presented with blasts in the cerebral spinal fluid at time of diagnosis. Treatment response of the CMMRD-associated T-LBL patients was favorable, as 75% achieved complete remission. In general, treatment was tolerated well in the CMMRD patients, without significant delay of chemotherapy. However, 3/8 CMMRD (37.5%) patients developed pulmonary aspergillosis infection during T-LBL treatment, which was fatal for one patient. This incidence is much higher than in the international EURO-LB02 study, where only 3/233 (1.3%, 95% confidence interval: 0.3–3.7%) of the T-LBL patients developed a pulmonary aspergillosis.

## CMMRD genetics

### Clinical description and first estimates of gene-associated cancer risk and survival in the context of CMMRD syndrome: a report from the C4CMMRD Database

Julie Robbe presented a study performed with Chrystelle Colas (Curie Institut, Paris, France) aiming to estimate the tumor risks in the context of CMMRD and to explore genotype–phenotype correlations. Data were extracted from the C4CMMRD Database and subgroup analyses were conducted according to the mutated gene and the variant type. One hundred CMMRD-patients developed 178 tumors (40% brain tumors, 31% hematological malignancies, 26% LS-related tumors and 3% of other type). The tumor spectrum varies according to the mutated gene. The median age of onset of the first tumor was 7 years (0.1–33.6 years) and was significantly different according to the germline mutated gene (*p* < 0.001). The cumulative risk (CR) of any tumor was significantly different according to the germline mutated gene (*p* < 0.0001) with the highest risk observed for biallelic *MLH1* patients (75% CR of cancers at 5 years of age). Survival was highly correlated to the mutated gene (*p* < 0.001): The median age at death was 6 years for *MLH1*, 7 years for *MSH2*, 13 years for *MSH6* and 20 years for *PMS2* biallelic PV carriers.

### Neurofibromatosis type 1 mosaicism in CMMRD patients

Establishing the differential diagnosis between CMMRD and Neurofibromatosis type 1 (NF1) can be difficult but remains crucial due to differences in treatment and surveillance [4, 7, 44]. Léa Guerrini-Rousseau (Gustave Roussy Cancer Campus, Villejuif, France) reported the case of a young girl with a clinical diagnosis of sporadic NF1 during childhood who developed a glioblastoma at the age of 16, which questioned the original diagnosis of NF1. Tumor characteristics (loss of PMS2 expression by IHC and ultra-hypermutated phenotype) led to the suspicion of CMMRD. Germline analyses identified two *PMS2* heterozygous PV and functional assays confirmed the diagnosis of CMMRD. An *NF1* PV (VAF of 20% and 9% in blood and saliva samples, respectively) was also identified confirming a germline mosaicism for NF1, in accordance with the patient phenotype (large plexiform neurofibroma of the thigh and freckling). A retrospective analysis of a French cohort, identified *NF1* germline mosaicism in 2/23 other patients with CMMRD, demonstrating that such an association exists [45]. This may suggest that early post-zygotic *NF1* mutations could play a role in the phenotype of patients with CMMRD and that this pathway may represent a new therapeutic target in some patients [45, 46].

### Preimplantation genetic testing for CMMRD in a consanguineous couple

Carole Corsini (Montpellier Hospital, France), presented the first French Preimplantation Genetic Testing (PGT) for CMMRD in a healthy couple where both partners carried the same LPV in *MSH6*. The *MSH6* LPV had been identified after the diagnosis of CMMRD in their son who developed a HGG at the age of 3 years. Because of the high level of consanguinity (double consanguinity) in this family, both couple members shared a “normal” haplotype in addition to the “disease-causing” haplotype. Thus, particular effort was made to screen a large number of loci on chromosome 2 across a broad range of coordinates around the *MSH6* gene to identify informative Short Tandem Repeats (containing at least 4 different alleles) for accurate haplotype detection in each embryo during PGT. A successful pregnancy was achieved following the transfer of a heterozygous embryo. After internal and national discussions and explanation to the parents of the pros and cons of revealing the fetus’ genotype, it was decided not to communicate that the fetus was an LS carrier at the time of successful implantation, but a future oncogenetic consultation has been recommended when the child reaches maturity. Following this presentation, several aspects of genetic counseling were discussed, in particular the advantages and disadvantages of informing minors who have undergone germline analysis to exclude CMMRD if they are carriers of LS, for which the oncological risk mainly concerns adulthood. Informing LS carriers who are of reproductive age about the risk of CMMRD for their offspring in cases of consanguinity or in populations with known MMR founder variants was addressed, as was the possibility of testing their partner before pregnancy.

## Closing remarks

The 6th C4CMMRD meeting covered a number of advances in the detection, management, and biological understanding of CMMRD. Guidelines for the diagnosis of CMMRD and CMMRD cancer surveillance protocols have been published from two expert groups, the C4CMMRD consortium and the International Replication Repair Deficiency Consortium (IRRDC) [3, 5, 7, 47–49]. The surveillance guidelines were recently evaluated by both consortia [10, 50].

The C4CMMRD Board highlighted that several C4CMMRD members were working with the European Reference Network (ERN) for all patients with one of the rare genetic tumour risk syndromes (GENTURIS) to update the guidelines on diagnosis and surveillance, and to formulate new recommendations on clinical treatment, quality of life and genetic counseling for CMMRD patients. The ERN GENTURIS guidelines take all new findings and advances which were presented to a large part at the 6th C4CMMRD meeting in to account and will be published soon.

## Data Availability

No datasets were generated or analysed during the current study.
